# Mental health professionals’ experiences of working with parents with psychosis and their families: a qualitative study

**DOI:** 10.1186/s12913-021-06416-1

**Published:** 2021-04-27

**Authors:** Jessica Radley, Jane Barlow, Louise Johns

**Affiliations:** 1 Department of Psychiatry, University of Oxford, Warneford Hospital, Warneford Lane, Oxford, OX3 7JX UK; 2 Department of Social Policy and Intervention, University of Oxford, Oxford, UK; 3 Oxford Health NHS Foundation Trust, Oxford, UK

**Keywords:** Healthcare professionals, Psychosis, Parenting, Qualitative, Thematic analysis, Focus group

## Abstract

**Background:**

Healthcare service users who are parents with psychosis form part of the caseload of most community mental health teams. Mental health professionals can experience uncertainty about how to work with and ask about the children of these parents, and often report difficulties when collaborating with other agencies. This study focused on professionals’ experiences of working with parents with psychosis and their families to gain an understanding of these parents’ needs from a service-level perspective, and to identify barriers that professionals may experience in meeting those needs.

**Methods:**

Qualitative focus groups were conducted with four to eight mental health professionals per group. Data were analysed using reflexive thematic analysis. JR familiarised herself with the transcripts and then coded each salient unit within the text. Themes were then identified and discussed amongst all authors until there was agreement.

**Results:**

We developed two overarching themes: 1) Diversity of need in parents with psychosis and 2) Role boundaries. The first explored mental health professionals’ perceived range of experiences that parents with psychosis and their families have, and the range of potential effects of parental psychosis on a child. The second theme described how some mental health professionals emphasised the importance of supporting service users in terms of their parenting status and others felt it was more critical to treat the person’s symptomatic expression. This theme also included issues with communication both with their service users and with other agencies.

**Conclusions:**

Mental health professionals identified that the needs of parents with psychosis were diverse and reflected significant variation in the experiences of service users. Mental health professionals across different types of team (early intervention and community mental health) expressed contrasting viewpoints about how achievable it was to respond to a service user’s parenting status in an adult mental health setting. Future research should aim to determine where training is needed to enhance mental health professionals’ ability to work holistically with families in an adult mental health setting, and how to enhance collaboration with other agencies.

## Introduction

Patients with psychosis make up over half of the service users in a community mental health team [1] and over a third of individuals with psychosis are a parent [2–4]. These parents are more likely than the general population to live alone [5], be unemployed [6] and require social service intervention [7, 8]. Furthermore, coping with psychotic symptoms and managing the side-effects of medication may result in parents becoming less emotionally responsive to their child’s needs [9, 10]. However, it is primarily patients who experience more severe psychotic episodes and have worse adaptive functioning who show a deficit in the quality of care they provide for their children, and the majority of parents with psychosis do not show any impairment in parenting ability [2, 11]. The children of parents in mental health services are often invisible as they are not routinely recorded on service users’ case notes [12, 13]. As a result, the ‘Think Family’ approach in the UK has called for improvement in the identification of these children and the signposting of families to other agencies [14].

For adult mental health professionals, working with a service user who is a parent brings the extra responsibility of ensuring the safety and wellbeing of the child [ren]. Evidence suggests that these professionals can experience anxiety in relation to their role with these children [15–17] and uncertainty about whether to involve the service user’s family in their care [18]. Some mental health professionals have also reported that they are wary of mentioning the service user’s child [ren] for fear of damaging the therapeutic relationship with the service user [19, 20]. These professionals feel that they are inadequately trained with regard to working with a service user who is also a parent [21, 22]. There can also be a lack of agreement about how much responsibility they have for the children vis-a-vis other agencies who also have a role in supporting the family [19, 23], with collaboration with these agencies often being seen as ineffective by those working in mental health services [13, 21].

Previous qualitative work has focused on adult mental health professionals working with parents regardless of diagnosis (e.g. [16, 24]). However, parents with psychosis have specific needs relating to their condition; for example, the acute symptoms of psychosis may mean children become involved in delusions and hallucinations [25, 26], and the episodic nature of psychosis may require different kinds of support for the family depending on whether the parent is stabilised or is experiencing acute psychotic symptoms. Working with diagnosis-specific groups has also been recommended by researchers [2, 27] and requested by the parents themselves [28].

This paper aims to: (1) understand the needs of parents with psychosis and their families from a service-level perspective; (2) explore the barriers that mental health professionals face when working to meet these needs. We used qualitative methods in order to explore the perspectives of mental health professionals and combined the viewpoints from two different kinds of mental health service.

## Methodology

### Design

Focus groups with mental health professionals who had ever worked with a parent with psychosis were conducted to address the study questions. Initial questions for the focus groups were designed after reading the findings of qualitative research conducted with similar populations (e.g. [15, 18, 29]). Aspects of grounded theory were used in the design such that question formation, data collection, and analysis were undertaken in tandem rather than consecutively [30]. For example, after conducting the first focus group, the researchers were interested in probing the topic of the importance of recognising a service user’s parenting status, and that area of discussion was prompted in the two subsequent focus groups. Data collection was conducted in focus groups of mental health professionals from different professional backgrounds who worked together and were familiar with each other. This format allowed discussion to take place easily between colleagues who shared the same working environment. Three focus groups were conducted, each comprising between four and eight participants.

### Participants

Participants were recruited from Early Intervention in Psychosis teams and Adult Mental Health Teams in Oxford Health NHS Foundation Trust. Participants could be from any professional background and were eligible to take part in focus groups if they had worked (i.e. visited at least once a month) with patients with psychosis who were also a parent of one or more children.

### Procedure

Two Early Intervention in Psychosis (EIP) Teams and four Adult Mental Health Teams (AMHTs) were approached. The researchers were able to arrange focus groups with three of these teams. Two groups were with the EIP teams (FG1 and FG2) and one with an AMHT (FG3). Each focus group included participants from a variety of professions who worked within the same team. The groups took place between June and November 2019. They were held in the office space of each team, and lasted between 45 and 70 min. JR began each focus group by asking each participant to state their name and role within the service. Participants were encouraged to speak to each other when discussing topics rather than solely addressing the researcher. Participants who had not contributed much at the beginning of the focus group were directly prompted for their thoughts in the second half of the session. The questions posed to the groups addressed staff perceptions about the needs of these families, service provision, and barriers to working with these families.

### Analysis

The audio-recordings of the focus groups were transcribed by the JR. We used reflexive thematic analysis [31, 32] to identify patterns within our data. The first author read over the transcripts to familiarise herself with the data while making notes of initial salient points. Then each meaningful unit of data was coded using NVivo 12 software. Coding began immediately after the first focus group was transcribed, which allowed the researcher to adapt the interview schedule to investigate important aspects highlighted through coding. Once all three focus groups had been conducted and coded, these codes were brought together to start the process of identifying initial themes and subthemes. These themes were checked and discussed with the other authors while referring to the initial codes and transcripts. Once the themes and their hierarchy had been discussed and developed further, they were named appropriately.

## Results

Three focus groups were conducted consisting of nineteen participants in total (see Table 1).

**Table 1:** Characteristics of focus group participants

	Focus Group 1 (FG1)	Focus Group 2 (FG2)	Focus Group 3 (FG3)
**Type of service**	Early Intervention Service	Early Intervention Service	Adult Mental Health Team
**Number of participants**	4	8	7
**Participants’ Profession and Gender**	2 Community Psychiatric Nurse (2F) 2 Social Worker (1F, 1 M)	3 Community Psychiatric Nurse (2F, 1 M) 3 Social Worker (3F) 1 Occupational Therapist (1F) 1 Psychotherapist (1 M)	3 Community Psychiatric Nurse (2F, 1 M) 1 Occupational Therapist (1F) 2 Psychiatrist (1F, 1 M) 1 Support worker (1F)

**Fig. 1:**
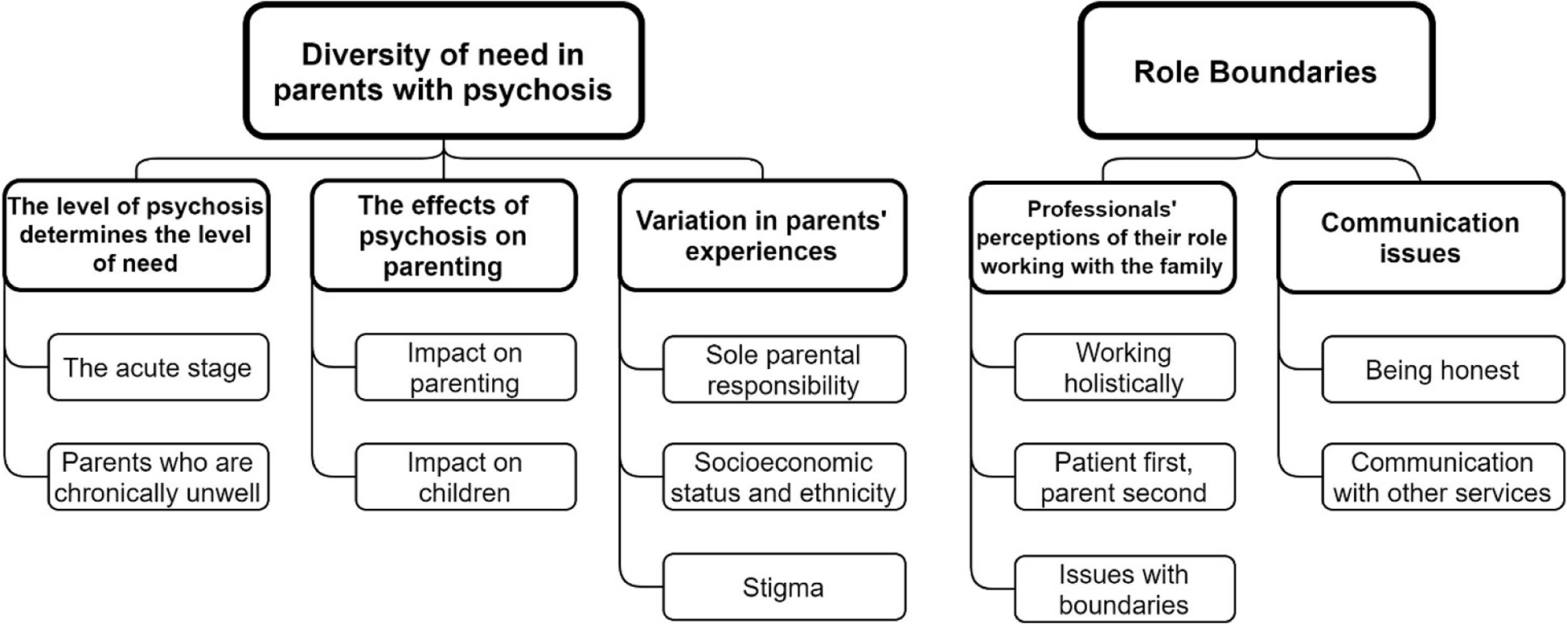
Themes and Subthemes

We developed two overarching themes: 1) Diversity of need in parents with psychosis and 2) Role boundaries (see Fig. 1). The first overarching theme describes how participants felt there were many factors that influenced what kind of needs a parent with psychosis and their family might have. The second highlighted how issues relating to communication, both with parents and other services, were viewed, in terms of the appropriateness of intervening with the whole family.

### Diversity of need in parents with psychosis

The first overarching theme demonstrated how participants felt that there was no ‘typical’ parent in terms of needs related to parenting. There were three subthemes 1) The level of psychosis determines the level of need, 2) The effects of psychosis on parenting, and 3) Variation in parents’ experiences.

#### The level of psychosis determines the level of need

This subtheme described how participants perceived a difference in service user need in their role as a parent when they were in an acute stage of psychosis compared to when they had stabilised. It also included their views about the impact of being chronically unwell on the service user’s capacity to parent.

##### The acute stage

Participants noted that when a parent was in an acute stage of psychosis, in which they were actively experiencing hallucinations and delusions, the participant would turn their focus towards the needs of the children in terms of being prepared to raise safeguarding concerns: *“Obviously if there are issues, whether or not you’ve managed to treat that person, the safeguarding, I think, overrides it all” (FG1, P02).* Participants described how there may be a contrast between rational behaviour in a stable state and risky behaviour in an acute state: *“I had a mother who I was working with, that happened* [sic]*, and she became acutely suicidal, became very very unwell*, *and needed to be hospitalised for several months. And safeguarding issues came up at that point because, obviously, trying to commit suicide when you have a young child in the house is high risky, but at the rest of the time she’s a very good mother” (FG1, P04).* However, participants noted that this acute stage often does not last long: *“Thankfully, acute floridly psychotic patients don’t stay like that. People tend to recover whether it’s with medication, whether it’s with time. People are very rarely floridly psychotic for a long period of time” (FG2, P05).*

When a parent had stabilised after an acute episode, participants felt they were then more able to engage with them as a parent on topics unrelated to safeguarding: “*… towards the end when people are getting better and feeling like* [sic], *we can do more and engage with them as parents rather than perhaps the safeguarding and other stuff” (FG2, P05).* The episodic nature of psychosis was also viewed as meaning that parents could be with their children in between these episodes: *“And the other thing is it’s an episodic illness so people can get better in between times and so there’s no reason why they shouldn’t be looking after their children in between times” (FG3, P07).*

##### Parents who are chronically unwell

Participants drew a contrast between the needs of parents who had recently experienced a first psychotic episode and parents who were chronically unwell: *“Our raison-d’etre is because it’s first episode, it may be unique in itself if you get the right treatment quickly enough. I think that’s the general ethos so that if we do the job that we’re expected to do and we have the means to do it, then there’s no reason why that person can’t recover and carry on with what they’re doing. Whether they’re good or bad parents is not the issue [ …*], *whereas if you were in a community team, an adult mental health team, where people are chronically unwell, I think that’s a different set of situations and different things apply” (FG1, P01).*

Participants within the AMHT were as such more likely than participants working within EIP teams to describe their service users as having experienced removal of their children or having less contact than they wanted due to the severity of their illness: *“I can think of someone very current at the moment where the welfare of her child is paramount and during her acute deterioration the child had to be removed” (FG3, P06).*

#### The effects of parental psychosis

This subtheme described participants’ views regarding the array of effects of parental psychosis on the service users’ ability to parent and also on their children, with participants being keen to highlight the fact that the diversity of symptoms experienced meant that their service users’ parenting wasn’t always affected: *“The symptoms may be different and it affects them in a different way and it doesn’t mean that you’re not capable of being a parent just because you have a psychotic illness” (FG2, P07).*

These effects were further described by two subthemes.

##### Impact on parenting

Participants described how anxious these parents were as a result of their condition, and how this anxiety can be exacerbated after experiencing a psychotic episode. Some participants hypothesised that such anxiety might be a prodromal symptom leading up to their psychosis: *“Before they’ve experienced psychosis they were sort of probably* [sic] *teetering on the edge of being sort of an anxious mum, and then they found the experience of psychosis just exacerbated that, and now they find they’re that sort of* [sic] *hyper-vigilant mum, where the kid says they’ll be back at half past five and at five thirty-five they’re calling and checking” (FG2, P06).* Participants described the parents’ feelings of guilt for not managing to parent well at all times as a result of their psychosis: *“I know I certainly can think of a number of parents who feel incredibly guilty and actually largely this guilt is of not being able to do the parenting role as well as they would like to” (FG1, P01).* These parents were also perceived to be self-critical and lacking in confidence even when, in reality, they were managing well: *“Apart from when someone’s acutely unwell, I think often they are people who are more sensitive to their children’s needs, and more prone to worrying about doing things effectively, and more prone to sort of be self-critical. So yeah I think probably high levels of worrying but actually often I think they’re doing things relatively well” (FG1, P04).*

Many participants described how these high levels of anxiety about parenting often persisted during psychosis and could manifest as paranoia, resulting in these parents taking actions to protect their child: *“I was talking to someone yesterday who was well and she was talking about how when she’d been unwell, she’d just wanted to protect her son. And that she was putting things behind his mirror and his wardrobe to protect him and to stop him being killed and stuff* [sic]*. It was all about protection” (FG2, P01).*

Descriptions of parents putting their children first, even when psychotic, included mothers who wouldn’t perform any personal care for themselves, but made sure their children were clean and fed and at school on time: *“As soon as they’d left the house then she’d slunk in* [sic] *the sofa until they came home again. And she never brushed her hair because it was always tied up, but the children always had immaculate clothes. The children were always fed. The children were always looked after” (FG2, P01).*

However, participants also described scenarios where the parent was so unwell they neglected their children’s needs completely: “*Usually the parent is paranoid; they’re usually terrified something is going to happen to their child. They’re scared for themselves and they’re scared for the children. You know, if they’re paranoid, that’s one aspect. The other aspect is you might have a parent who’s so unwell that they neglect their children because they’re unable to look after the children’s needs. It varies” (FG2, P01).*

Fatigue and weight gain associated with antipsychotic medication usage was perceived by participants to contribute to a lack of motivation to engage with their children: *“Chronic problems are just around sedation, weight gain, kind of lack of energy. Those are very much the things, you know. They can’t take their children to the park or wake up in the morning and that’s quite destructive in the long term” (FG1, P04).*

##### Impact on children

Participants described how the impact of psychotic symptoms on parenting in turn impacted on the children of these parents. There were frequent reports of the paranoia experienced by parents resulting in the children having fewer opportunities to leave the house, for example to go to the park, because their parent was too frightened about what might happen. Participants described how these paranoid experiences had made some parents anxious for the safety of their children: *“The difficulty when the parent teaches the child that it’s a very dangerous world out there, and there are dangerous people there, and you can’t trust anyone. ‘Don’t tell anyone because social services will take you away’” (FG2, P01).* Other ways in which children’s freedom was impacted were also described: *“‘Mum cleans the floor. She’s quite obsessive. She thinks there’s contamination’. They’d have to sit on the settee for hours because she didn’t want them to dirty the floor again cos* [sic] *she’s just cleaned it” (FG2, P03).*

Participants identified many instances where children had experienced trauma after witnessing events caused by their parents’ psychosis, and some had begun to exhibit behavioural issues: *“They’ve seen the trauma and the police coming in so things like that. They don’t know why it’s happening and now you’re unwell, and they don’t know to what extent, and they don’t want to make it worse. So it is a very tricky situation and to get social services involved that is scary in itself for the children and family. They’re scared they’re going to be separated” (FG2, P03); “Two of them are attending school and their behaviour has changed adversely, their attendance to classes has dropped, and they have, perhaps, not been as able to carry on as they were before their father had this episode” (FG1, P01).*

Some children were described as trying to avoid the parent or being embarrassed witnessing their parents trying to cope with symptoms*: “The parent is in the supermarket trying to cope with voices and the kid is really embarrassed” (FG2, P08).* The children of these parents experience further anxiety when they realise safeguarding concerns have been raised and fear potential removal from their families: *“I know the children at the meeting I was at last night, they were really really anxious. I mean it was done in the family’s front room to try and be* [sic] *a more relaxed environment. They were really quite agitated by us all being there despite trying to make it very relaxed and I think it was a sense of ‘ooh there’s a real problem, things are wrong’ despite the fact we tried to be reassuring” (FG1, P04)*. This participant also described how children would minimise their problems *“cos* [sic] *they’re so anxious. Certainly the children last night were very anxious about us being there and were very much saying ‘oh everything’s fine there’s no problems cos* [sic] *everything’s lovely’” (FG1, P04).*

#### Variation in parents’ experiences

Participants reported that a range of factors influenced how parents managed episodes of psychosis, and how services responded to their need.

##### Sole parental responsibility

Some participants believed that single mothers with sole parental responsibility were targeted more for child removal by social services: *“Unfortunately it’s usually the single parents … it’s usually mothers who don’t have anybody around them who can sort of step in. That’s usually when social services go in a lot harder and that’s when the mothers normally need more support and in that situation, they often do end up losing their kids” (FG2, P05).*

These mothers were often said to be isolated because in addition to having sole parental responsibility, they were sometimes managing experiences such as paranoia and anxiety. It was noted that these mothers would most likely benefit from meeting other mothers with psychosis in some kind of peer support setting where they could “g*et support from the family centres and not be isolated and meet other mums and things like that (FG2, P01)”.*

##### Socioeconomic status and ethnicity

The relationship between other factors, such as social circumstance and ethnicity, and concerns about child protection were also highlighted. Participants perceived disadvantaged families as being more likely to have their children removed by social services: *“Social services do nothing because these are parents who can write good letters whereas have they been* [sic] *from a working class background, an ethnic background, those kids would have been in care one hundred percent” (FG2, P05); “Social services weren’t interested because the husband’s a doctor” (FG2, P01).*

##### Stigma

Staff also felt that stigma towards mental health diagnoses such as schizophrenia made it more difficult to regain parental rights: *“She’s going through court at the moment trying to get her time increased with her daughter because there’s that stigma … the fact that she’s got schizophrenia. Her ex-husband is very cautious about increasing that contact because there’s the fear that you’re not fully recovered or something will happen” (FG3, P03).*

### Role boundaries

The second overarching theme described the range of beliefs that participants held in relation to their role and included two subthemes: 1) Professionals’ perceptions of their role working with the family and 2) Communication issues.

#### Professionals’ perceptions of their role working with the family

A range of views was expressed regarding the importance of taking parenting status into account when working with a service user. This is explored in the subthemes below.

##### Working holistically

Some participants believed that it was important to work as holistically as possible at all times, and this included recognising a service user’s status as a parent, especially in certain circumstances, such as single parenthood: *“… then yes you have to care about their illness but you almost instantly have to care about the fact that they’re a solo parent” (FG3, P06).*

Participants talked about finding a way to develop a relationship with the child, which was sometimes independent of their parent: *“‘What did you do at school today?’, ‘what did you have for dinner?’ … there’s obviously building rapport with the family and just being polite but also gives me some information about have they had their dinner or how are they doing? Do they seem happy and well in themselves as well?” (FG2, P06); “He’s aware of mum’s struggles and, bless him, he emails me every now and again just to get a bit of an update and I think that’s been really helpful for him” (FG1, P02).* They also described how their input with the children was adapted to address their developmental needs, such as for example, when providing them with information: *“It’s got to be age appropriate so it depends on what you say. I mean if the child’s fairly young and the parent’s experiencing it in front of them, they’ve got to at least understand that ‘we think your parent is unwell’ but not necessarily go any further. Whereas when they’re much older you can explain a bit more” (FG3, P06).*

Some participants even noted that the children may notice warning signs that the parents themselves do not, and therefore it may be helpful to involve older children in their parent’s care: *“They [the children] give quite an honest overview of ‘okay well I notice mum doing this or dad doing this’ when perhaps they [the parent] didn’t realise they were doing that themselves. So if it’s appropriate, they can almost be involved in that” (FG1, P03).*

Some participants also perceived their role to include working to reassure parents: *“If someone’s anxious because of the impact the mental health is having on the parenting ability … it’s kind of relaying that ‘that just shows us how much you care about your children’” (FG2, P06).* They emphasised the importance of showing the parents on their caseload that they respected their role as a parent, even if the child had been removed: *“I think we have to be also very very mindful to respect them as a parent no matter* [sic] *regardless of whether or not they’re still parents. They’re more than just the psychiatric diagnosis if you’d like” (FG3, P06).*

One participant described how their location within the health care system (i.e. EIP or AMHT) affected their ability to work with the service user in this way: *“I reckon on the whole in early intervention we probably have more capacity to do that kind of monitoring. Our caseload is usually half what an AMHT is so I guess if this is a conversation among AMHT clinicians, they would probably struggle” (FG2, P05).*

##### Patient first, parent second

Some participants felt that their role should be focused on the service user. They perceived it to be unrealistic to have a relationship with the children of their service users, in addition to the service users themselves. This was partly to do with time constraints, but also because of limited training: *“I think before all of that you really do need to have some training, some sort of expertise learning in order that* [sic] *you don’t go in with the best intentions but actually cause more harm than good so I think it has to be structured. It has to be quite meaningful” (FG1, P01).*

The participants who expressed this view explained that other services were better placed than they were to work with these children, and that their role was to work in collaboration with such agencies: *“We’re not able or skilled or indeed briefed to be more inclusive of all the family, so we tend to be focusing on that individual and that’s what we’re there to do, that’s where the emphasis of what our work is meant to be placed on. But I think it really is important to work with other agencies if they’re involved and they’ve been identified” (FG1, P01).* These participants identified the need to have another professional looking after the needs of the child: *… and that’s where perhaps having somebody that’s speaking from the child’s perspective and have them as the priority as opposed to us having our patients as being our priority” (FG1, P02).*

These participants felt that it was important to treat their service users as ‘patients’ first and parents second. They perceived the children’s needs as being best served by them, as the mental health professional, focusing on helping the parent to recover from their mental health problem, thereby enabling them to resume their parenting role: *“It’s generic what we do and we might be dealing with someone who is our patient who happens to be a parent, or who is our patient who happens to be a doctor, or who is our patient who happens to be homeless. So actually being a parent, it’s not secondary but it is secondary to what we are dealing with” (FG3, P05); “They’re with us with a view to help them address their symptoms and be able to recover so their primary need is to get well and obviously we expect that if we can achieve that, then they will be more able to parent their children successfully” (FG1, P01); “Our first and foremost is about the individual who has been referred to us or who is at the centre as a patient before as a parent” (FG3, P01).*

Another participant described how she had believed herself to be working holistically until attending a funeral of a service user, where she met the family and realised that she had not truly accounted for all parts of this person’s life: *“I did try to deal holistically with this patient but actually I went in as the mental health nurse, made sure she’d had her injection, made sure she was in, made sure she was concordant, made sure that her mental health was stable, and that’s just a small part. Her funeral made me realise she’s far more than just someone with a mental health problem” (FG3, P05).*

##### Issues with boundaries

Some participants indicated uncertainty with regard to where the boundaries of their role with the families lay, and specifically in terms of their role with the children of service users: *“… cos* [sic] *I do find it’s sometimes a bit of a grey area. I’m seeing a patient who’s a parent and she’s got two young people both with special needs and where is my role in that? Obviously I’m here to support her, but obviously to support her I need to support her children, but also not having enough information and knowledge just generally about what that would involve at the time. But she was having meetings at school with the daughter and I eventually did get involved, but to start with it was kind of you know ‘do I?’” (FG1, P03).*

Another participant noted that there were times when she was having to balance the safeguarding needs of the child with concerns about exacerbating problems for the parent: *“You’re trying to find that balance all the time between acting safely and not overly sort of* [sic] *escalating things because even if it’s intended to be protective, it can really increase anxiety levels” (FG1, P04).*

#### Communication issues

##### Being honest

Many participants described how important they thought it was to be completely honest with service users about service expectations from them as a parent. They explained how they were transparent about the ramifications of not meeting expectations and making it clear from the initial meeting that if they did witness anything that caused alarm, they would be raising safeguarding concerns: *“I said ‘if you’re going to try and kill yourself in the house and you’ve got a young child in there, then that is going to lead to kind of major issues and you will not be able to maintain your role as a parent because it’s just not safe’” (FG1, P04).*

They described the importance of such communications being uncritical, and of framing any reference to social services as an additional support as opposed to judging their adequacy as a parent: *“Referral is not about them being judged as a parent. It’s for them to be supported as a family so I always put an emphasis on that to try to reassure to a certain amount as well” (FG3, P03); “‘We’re not trying to take your children away. That’s the last thing we want. We’re trying to put in extra support to enable you to continue to care for your children.’” (FG2, P07).*

Furthermore, there were descriptions of parents receiving social services positively and parents who missed the support once it was stepped down: *“With the support she found so helpful* [sic], *then when it came time to be stepped down, discharged, as it were, from social services she was actually sort of thinking ‘oh I’ve got a bit less support now’ and she was kind of wishing it could stay around for longer, which I was really surprised about” (FG2, P06).*

However, when prompted by the researcher, participants also described situations in which communication about the service user’s children was difficult or not possible. For example, some parents were secretive and wary of talking about their children, and participants described some parents as being reluctant to engage at all: *“I’ve certainly had people say you know ‘I don’t want any involvement you’re not entitled to that part of my life’” (FG1, P04); “I think possibly that comes from a distrust of services. Not understanding that social services isn’t just about taking the children away from families but can also offer support” (FG2, P05).*

Some participants described how, in certain circumstances, they were not completely honest about raising safeguarding concerns due to the nature of psychosis, and the increased likelihood that the parent may become paranoid or may be lacking insight into their own behaviour: *“I can think of circumstances particularly with people who are paranoid, and maybe it’s affecting the welfare of the child, and they’re just not going with what you’re suggesting, and the child is at a certain risk, then I think you might not necessarily involve the patient at that point like ‘by the way I’ve been talking to three different other* [sic] *agencies about you’” (FG3, P05).*

##### Communication with other services

Communication issues extended to the services participants were liaising with. Some participants, for example, perceived that social services did not always engage with them and that, when they did, they frequently exhibited over-reactions to the diagnosis of psychosis: *“Sometimes trying to get social services to do anything is the problem. To put anything in is … that sort of is the battle. You feel like you’re battling with the client against social services to put anything in to actually support … to look after the child” (FG2, P01); “There’s a lack of understanding from social services about mental health so the word ‘psychosis’ is a bit scary and everybody panics and get hung up on ‘psychosis’” (FG2, P07).*

Participants also felt that when social services were involved in the safeguarding of a child of one of their service users, they were overly focused on the parent’s diagnosis and expected them, as the mental health professional, to be involved at all times as their mental health worker, even when the parent’s mental health difficulties were no longer an issue: *“I think the problem comes when they start to have the multi-disciplinary meetings in social services and then we find that their mental health is stable and we want to withdraw and then there’s panic because we’re involved even though actually there’s no role for us anymore” (FG3, P06); “And actually we in mental health tend to be sort of looked to by other parties involved with children as the sort of … the main point of responsibility which isn’t always particularly correct but that’s how it is” (FG3, P06).*

## Discussion

### Key findings

This is one of the first papers to examine the views of adult mental health professionals, with regard to the needs of parents with psychosis and their families. Two overarching themes were identified from this reflexive thematic analysis: ‘Diversity of need in parents with psychosis’ and ‘Role boundaries’.

The first theme demonstrated that participants felt it was not possible to define the specific needs of parents with psychosis due to the diversity of this group of parents. While some parents were perceived to cope well with their parental responsibility or had good support systems to aid them, others had neglected their children and needed more involvement from services. Thus, while participants identified that a service user experiencing acute symptoms made intervention with the children more of a necessity, they also described how acute symptoms could be fleeting, and with a good support system, not require any further intervention. There was also a perception, that for some parents, their greatest need in relation to parenting was for recognition that the anxiety they were experiencing was understandable, and for reassurance in terms of their capability as a parent. A qualitative study in Sweden, which looked at mental health professionals’ perceptions of service users’ quality of parenting [33], also identified anxiety, paranoia and fatigue to have negatively impacted on parenting, although, in contrast to the current study, cognitive impairments and difficulties in empathising were also identified [33].

The second theme showed that within one team, professionals may hold differing views about the appropriate level of intervention with the family, and also highlighted communication issues for participants both with parents and other services. For example, some participants believed that service users were ‘a patient first and a parent second’, and although all participants affirmed that the child’s needs were paramount, this sub-group saw time as being a barrier to further involvement. They identified other services as being more appropriate to work with the families of these service users. In contrast, some participants strongly emphasised the importance of working holistically in terms of seeing the service user’s role as a parent as being central to their treatment. Some of these participants demonstrated how they had gone beyond what would be expected of them in their role in terms of the support that they provided to their service user’s children. They also believed that children could have a part to play in their parents’ recovery since they believed children were adept at identifying signs of relapse, although they recognised this involvement should be age-appropriate. Involvement of the children in their parents’ recovery could mean explaining the parent’s mental illness and including the child in certain elements of the recovery plan [25]. Any involvement, however, should not lead to them taking on caring responsibilities, as has been described to happen amongst children and adolescents living with parental mental illness [34, 35].

This contrast in viewpoints with regard to working holistically is consistent with other qualitative studies conducted with mental health professionals working with parents with a mental health diagnosis, which also found differing viewpoints within their sample of professionals [16, 23, 36]. Specifically, two studies found that mental health professionals differed in terms of whether they focused on the welfare of the child or the parent [16, 20], one study highlighted the reluctance of some mental health professionals to engage in family-focused practice [36], and another study showed disparity in terms of whether mental health professionals perceived themselves to be responsible for the delivery of parenting interventions [23].

However, it should be noted that the ‘patient first parent second’ group of participants largely viewed the parental role as being secondary due to a lack of time, training, and the belief that other professionals may be more appropriate to address the family’s needs. Indeed, one participant stated his concerns of doing more harm than good without adequate training. To make any change or innovation within a healthcare setting, the established beliefs of the healthcare professionals who are expected to implement this change must be taken into account [37]. Burnout is common in mental health professionals [38], and factors such as excessive job demands and working in a community mental health team are more likely to result in lower morale [39]. This points to the need not to put further demands on mental health professionals to go beyond what is expected in their role, but rather to move towards an approach where mental health professionals feel they have the training and capacity to support their service users holistically to aid recovery.

Researchers have called for improvement in the reporting of parenting status in mental health services [12, 13] and the Think Family approach in the UK has reflected this [14]. Some countries have made legislative changes to increase identification of children [40–42]. Additionally, parents themselves feel that recovery and parenting are intertwined [43] and interventions are increasingly being designed to treat parenting as a central element to recovery [42, 44]. Research suggests that care should be family-focused and recovery-oriented and in order to achieve this, whole families must be put at the centre of the individual’s care alongside an increase in the capacity of service providers to do this [45, 46].

Some participants reported friction when working with social services, who they felt did not always communicate effectively with them as the lead mental health professional. They also thought social services could show bias against parents with mental health problems, often assuming intervention with the children was necessary solely because of the parental diagnosis. Participants felt that too much was expected of them in multidisciplinary meetings. In turn, previous research has shown that social services practitioners find collaboration with mental health services difficult, and that mental health workers were perceived to be overly focused on the psychiatric condition and reluctant to collaborate [24]. It is of the utmost importance that agencies can work together effectively in cases like parental psychosis where multiple individuals are involved and may have different needs.

### Strengths and limitations

A strength of this study was that it combined the perspectives of mental health professionals with different professional backgrounds across two types of team. Most research conducted with mental health professionals working with parents with a mental health diagnosis does not focus on psychosis but rather a range of diagnoses (e.g. [16, 19, 47]), and, when it does focus on psychosis, it usually involves professionals working with mothers experiencing postpartum psychosis (e.g. [21, 22, 48, 49]). This study had a specific focus on parenting in the context of a parental diagnosis of psychosis, and one of the subthemes, has provided important information about how mental health professionals view psychotic symptoms in terms of their impact on parenting.

A limitation of this study was the lack of time to conduct additional focus groups. The possibility of a contrast between professionals from Early Intervention in Psychosis Teams and Adult Mental Health Teams was suggested by the researchers and even by some professionals within EIP teams during the focus group, although in actuality there was consistency in the data across each focus group for each subtheme. With more time and resources, it would have been interesting to conduct another focus group with an AMHT to further explore potential differences between the two types of team.

### Implications for practice and future research

The Think Family approach suggests that when working with families with parental mental illness, there must be accurate recording of children, appropriate signposting to other services, improvement in multi-agency collaboration, involvement of children in care planning where appropriate, and that the needs of each individual in the family must be taken into account at assessment [14]. This approach also emphasises the importance of senior managers supporting their staff and providing appropriate training to enable them to achieve this [14].

Despite this, the current study identified uncertainty about the level of involvement a mental health professional should have with the children of their service users, and difficulties in collaborating with social services. Some participants lacked knowledge about the Think Family approach [14]. These participants felt they did not have adequate capacity to holistically support families on their caseload, and a need for more education and training was identified. Future research should examine the specific knowledge gaps professionals have in relation to working with the children of their service users, and what training is needed to address this.

### Reflexivity statement

JR conducted the focus groups and it would have been clear to the participants that she was younger than most of them. Although she was known to some participants through contact about other research projects, she was not known to most. While the intention behind the research was explained at the beginning, one participant from one focus group implied on a number of occasions during the discussion that s/he felt there was a particular answer JR was looking for. The format of having colleagues in the focus group meant participants were comfortable discussing topics that were familiar to them and there were many examples of participants building upon each other’s answers. However, there were some signs of tension within one of the focus groups potentially due to strained professional relationships. For example, the conversation in that focus group became heated towards the end when one participant criticised another for using anecdotes rather than ‘evidence’ to support the point they were making. When this happened, JR introduced a new question into the group, which was directed to different participants.

## Conclusions

The results from these focus groups indicate that participants viewed the needs of parents with psychosis as being dependent on the level of psychotic symptoms, the parent’s contextual environment, and the presence of support systems, for example a partner with whom to share parenting responsibilities. This study also demonstrated that a range of viewpoints can exist within one team in terms of how much intervention is necessary, or possible, with a service user who has parenting responsibilities. Collaboration with other agencies was seen as potentially beneficial but many participants reported communication issues when doing so. More work is still needed to ensure that mental health professionals are given the necessary training and support to be able to work holistically with service users who are parents.

## Data Availability

The data that support the findings of this study are available on request from the corresponding author JR. The data are not publicly available as they contain information that could identify the research participants.

## Abbreviations

AMHT: Adult Mental Health Team
EIP: Early Intervention in Psychosis Team
FG: Focus group
